# Data warehouse for assessing animal health, welfare, risk management and –communication

**DOI:** 10.1186/1751-0147-53-S1-S3

**Published:** 2011-06-20

**Authors:** Annette Cleveland Nielsen

**Affiliations:** 1 Danish Veterinary and Food Administration, Unit of Veterinary Epidemiology and Health Management, Center for Veterinary Disease Control, Animal Welfare and Trade, Mørkhøj Bygade 19, DK-2860 Søborg, Denmark

## Abstract

The objective of this paper is to give an overview of existing databases in Denmark and describe some of the most important of these in relation to establishment of the Danish Veterinary and Food Administrations’ veterinary data warehouse. The purpose of the data warehouse and possible use of the data are described. Finally, sharing of data and validity of data is discussed. There are databases in other countries describing animal husbandry and veterinary antimicrobial consumption, but Denmark will be the first country relating all data concerning animal husbandry, -health and -welfare in Danish production animals to each other in a data warehouse. Moreover, creating access to these data for researchers and authorities will hopefully result in easier and more substantial risk based control, risk management and risk communication by the authorities and access to data for researchers for epidemiological studies in animal health and welfare.

## Introduction

Denmark has over the years established many databases concerning veterinary husbandry and animal health and welfare related data, both as industry and government owned databases. Examples are the Central Husbandry Register, (CHR), [1], gathering stem data on herds, VetStat [2,3], the antimicrobial database and VetReg [4] the veterinary practitioners register.

In order to have easier access to data and relating data from various databases to each other, the Danish Veterinary and Food Administration (DVFA) is establishing a veterinary data warehouse, which should be fully operational in 2012. The data warehouse should hopefully be of benefit for both authorities and researchers in their control purposes and research, respectively.

The government owned databases are legally based and therefore consisting of national data covering the total population of production animals, whereas industry owned databases often consist of voluntary data and are therefore often only covering a subpopulation and cannot be used for governmental purposes on a national scale. Due to this fact and legal concerns on gathering data from privately owned databases, the data warehouse will, at least initially only consist of government owned data. In Denmark, traditional risk analysis concerning food safety is divided into the following three parts: 1) risk assessment, which is done by the Danish Food Institute at DTU (the Danish Technical University) 2) risk management done by DVFA and 3) risk communication, which is also done by DVFA, communicating the risk management strategies and expected results to all interested parties. The many databases in Denmark are used for this risk analysis and so will the data warehouse be.

The data warehouse is part of a political decision by the Ministry of Food, Agriculture and Fisheries to optimize welfare in the Danish swine and cattle production.

## Data in the warehouse

Many of the data in the warehouse originate from databases already owned by the Ministry of Food, Agriculture and Fisheries, but new data will also be integrated in the warehouse, especially data related to welfare.

Already existing databases, which will be integrated in the warehouse are: CHR, VetStat, the Veterinary practitioners register, VetReg, the Control data register - from inspections in food and animals, the Laboratory tests register (national mandatory tests), the Zoonosis register (ZOOR) with data on Salmonella seroprevalence in swine, the Poultry database with serology and ante mortem samples for Salmonella, the Meat inspection database for cattle and swine and BSE and TSE databases.

### The Central Husbandry Register, CHR

All holdings with cattle, pigs, sheep and goats and also commercialised holdings with poultry, fur animals, deer, game birds and fish are registered in CHR [1]. Data are stem data on herd identification, farmers’ and veterinarians’ name and addresses, type and size of herds, location with GIS coordinates (Geographical Information System) and movement of swine, cattle, goats and sheep etc. The data are on the herd level for swine and other animal species, whereas they are on animal level for cattle. Especially birth, death, breed and movement are also registered for cattle on the animal level. For each batch of pigs being moved, the following information is recorded: the number of pigs, the date and time of shipment, holding of origin, destination of the animals and registration number of the vehicle used for the transport.

### VetReg

The national VetReg database [4] has identification of all vets and practices and these data are for instance used in order to keep track of all veterinarians, when disease outbreaks occur and are regularly used in DVFAÂ´s supervision and medicine control of veterinarians and in the mandatory veterinary advisory service contract between the farmer and veterinarian [5].

### VetStat

The national VetStat database [2,3] registers all use of antimicrobials for production animals on herd and veterinary level and on animal species and age groups. Every prescription is registered with identification of veterinarian and herd, drug identification, quantity of antimicrobial and disease group. Animal Daily Doses (ADD) are used in order to estimate the consumption. ADD is an assumed average daily dose per animal, defined as the daily maintenance dose for a drug used for its main indication in a specified species. The dose is defined for a standard animal”, i.e. an animal with an estimated average weight within a specified age group. In VetStat, ADDs are calculated for each age group [6]. The Danish Integrated Antimicrobial resistance Monitoring and Research Programme (DANMAP) [7] monitor consumption of antimicrobials from VetStat data and investigates associations between use of antimicrobial agents in animals and humans and occurrence of resistance among bacteria from animals, foods, and humans. Other countries as Norway, Sweden and the Netherlands also report on veterinary and human antimicrobial consumption. Some report wholesale data and other report data from pharmacies. Examples are NORM/NORM-VET [8], SVARM [9] and MARAN [10].

### Meat inspection data

Meat inspection data on the animal level for cattle and swine will also be part of the warehouse and from these, registrations related to welfare or infections can be found. For example weight of sows and registrations for emaciation are recorded, as well as hernias or infectious related registrations as pericarditis and osteomyelitis. These data have previously been used to estimate health and welfare issues in swine [11-14].

### Welfare control data

A new database on decubital ulcers of the shoulder region of sows is being established and will also be a part of the data warehouse. These data will be entered from several sources: a) as part of veterinarians’ registrations in the new mandatory veterinary advisory service contract b) as part of registrations in the farmers’ own new mandatory welfare program and c) at assembly plants for export of animals.

Data originating from DVFA´s annual welfare control on the herd level of five percent of all type of animal holdings will also be part of the warehouse. Likewise, data from the police on fines and court decisions on penalties regarding welfare issues will be part of the warehouse. From these, the most common irregularities from the welfare control can be drawn.

### Data on mortality

Data on mortality in cattle and swine herds will be calculated in the warehouse from the CHR register. The register holds data on birth and death of cattle on the animal level. For swine, movement data to rendering plants are used to calculate mortality in swine on the herd level.

### Future data

Examples of private databases owned by the agricultural sector are: a) The cattle database consisting of production registrations, mastitis control data and data on disease registration on the individual cow level and b) herd level swine production data, which for now are gathered on a regional level by the Danish Agricultural Advisory Service (DAAS). These data could be part of the warehouse in the future, as especially health and production data would be valuable in both studies of health and welfare and in risk management and control.

### Purpose of the data warehouse

Generally, easier access to multiple data from many databases will be essential in both risk management and risk communication, as well as for control and research purposes.

Control using only electronic records and registrations from the warehouse will result in easier preplanning and the later analysis of control data.

The purpose of the newly established Danish Centre for Animal Welfare (DCAW) [15] is to ensure, that research results and information from various relevant databases concerning animal welfare are collected and communicated to relevant stakeholders, including researchers. The data warehouse is a valuable tool for that purpose. DCAW has been established within the DVFA with representatives from universities in the board. The centre looks forward to collaborate with other institutions working with welfare issues and research.

When the warehouse is fully developed in 2012, exploratory data analyses and data mining for example could be applied on the data, describing underlying latent factors. This has previously been done on meat inspection data with the objective to describe health or welfare problems in swine herds [11-14]. This type of approach, using many data from many different sources – meat inspection, previous control data, antimicrobial consumption, data on decubital ulcers of sows, lack of ear tagging and mortality data – might identify hidden structures in data, which can be used to describe welfare and/or identify herds for risk based control. Both researchers and the welfare control by DVFA could benefit from establishing a welfare index for herds, using many different data from the warehouse. In addition, control data, for example on herds, where irregularities in the welfare control have been found, could be used for identifying data warehouse data describing these herds and after a factor analysis using control data, newly found factors describing herds with poor welfare could be used in DVFA's risk based control.

Geographical location and historical data on both animal and herd level will enable researchers to do studies on data distributed both in time and space, as for instance modelling spread of disease or other hierarchical data analyses.

Data from the warehouse could be used in prevalence studies on the herd level and national level and for creating threshold values in animal health or welfare. So far, DVFA has used data from VetStat and from CHR to establish national threshold values on the herd, animal species and age group level for antimicrobial consumption and mortality in swine and cattle. These threshold values are used in DVFAÂ´s risk management of antimicrobial consumption and mortality in herds.

When the warehouse is fully established in 2012, herd and national threshold values for prevalence of decubital ulcers of the shoulder region of sows and leg and hoof lesions in cattle will also be established.

DVFA is already using VetStat and CHR data and data from VetReg in risk management and risk communication of antimicrobial consumption, for instance in our supervision of all veterinarians on prudent use of antimicrobials and welfare control in herds. Standardized graphical displays of the veterinarians’ prescription patterns are for example used in risk communication in order to motivate veterinarians to a prudent use of antimicrobials. DVFA also use VetStat data for evaluating the effect of risk management strategies and risk communication.

### Sharing of data

There will be full data protocols for every variable in the data warehouse enabling users to understand and work with the data in spite of lack of previous detailed knowledge of the data within the warehouse. These data protocols will be public and can be used by all control personnel, farmers, veterinarians, researchers and others. Agreement on all data protocols and entries to the data warehouse has been established within the Ministry for Food Agriculture and Fisheries in order to have common knowledge and information on the data.

Many of the data related to the farmer's own herds (such as CHR and VetStat data)are already accessible on the internet both for the farmer and for the herd veterinarian, who holds the mandatory veterinary advisory service contract.

In the future, it might be possible for veterinarians and farmers to use the warehouse and look up antimicrobial usage and prevalences of welfare indicators etc. before, for instance, herd health visits, or the warehouse can be used as a management tool for the farmer, thereby increasing the awareness of welfare issues and hopefully optimize welfare status of herds.

### Validity of data

As no database and data warehouse is better than the data within it, the validity of data in the data warehouse is very important and several different strategies will be used to ensure the validity of the data. For example: continuous reporting of data, systematic control of entries and irregularities and cross-control of data with other sources. The validity of data will be controlled before entering in the warehouse. For example CHR contains automatic procedures for follow-up on missing, inconsistent or late notifications.

As control strategies often use herd size in calculations, for instance for antimicrobial consumption, prevalences of mortality or shoulder lesions of sows, it is essential that herd size is valid in the warehouse. This can be validated in several ways and herd size for a pig herd will here be used to demonstrate this. Initially, herd size is reported by the farmer to CHR. The farmers are required to have an up-to-date registration of swine in the herd and printouts are sent to the farmers on the registered data, so the farmer can correct discrepancies. The data on herd size can be cross-controlled by using data from many other sources, for instance the Plant Directorate. They do environmental manure control in herds and regulate EU funding to farmers. If the farmer reports too many swine in order to lower prevalences of diseases or antimicrobial use, then he risks reporting too many swine according to his environmental manure control in the Plant Directorate, as a higher number of swine will require more land. Cross-control on herd sizes can also be done using the movement database or the ZOOR register where all slaughtering is recorded based on the payment to the farmer. All these possible cross-control data are within the warehouse and can easily be used to cross-control herd sizes.

Other systems enhancing the validity of data are: control of data entry possibilities, repeated reporting of data, where inconsistencies are easier spotted or built–in systematic data control with reporting of discrepancies.

## Conclusions

Establishing a data warehouse gathering many of the data from existing Danish databases on animal husbandry and animal health and welfare should give easier and better access to interesting data for both researchers and authorities for epidemiological studies in animal health and welfare. The data warehouse will be of great value for DCAW in pursuing its goal about ensuring, that research results and information from various relevant databases concerning animal welfare are collected and communicated to relevant stakeholders. And DVFA's risk management, risk communication and risk based control will benefit from this collection of relevant data in the warehouse.
